# The Medical Buccaneer

**Published:** 1894-02

**Authors:** 


### *EDITORIAL.*

The office of THE JOURNAL is in room 330 Equitable Building.

Address all communications, and make all remittances payable to THE ATLANTA MEDICAL AND SURGICAL JOURNAL, Atlanta, Ga.

Articles for publication should reach this office not later than the 15th instant.

The editors are not responsible for views expressed by contributors.

### THE MEDICAL BUCCANEER.

This is the title of an editorial in the *Medical and Surgical Reporter* of January 27th, 1894.

It sets forth that until the early part of the present century the high seas were everywhere infested by bands of pirates in quest of prey. And further states that they were a thousand times more manly than the modern *medical* buccaneer.

The remarks touching this element in our profession are so well timed that we lay them before our readers as follows:

"To talk of amending codes, or instituting reforms, or improving the status of the profession while this insatiable gormand is allowed unfettered liberty is a travesty on reason. He plays his part in manifold ways. He often roams in high places, and may even wear a professor's gown. He looms up at medical conventions, and, indeed, may be an author of no mean position. He is always clamoring for reform; he wants to reform the code, let down the bars and clear the way so that his pilfering career may be unhampered. His neighbor stands in mortal terror of him, because he well knows that should he be required to call him in consultation the new arrival would quickly oust him and coolly take possession himself. He performs impossible operations, and always cures every case, and the unsuspecting, simple-minded, honest plodder, as he reads his statistics, is quite overcome with amazement and admiration. He has a sneaking way of advertising. To go into the regular column of the quacks would be to mix with the common herd; moreover, it is highly expensive; therefore he has himself *interviewed*, or one of his helpers will see to it that while the great man speaks full stenographic notes are taken, and the thing, highly colored, will be spread broadcast in the early morning press.

"The drag-net of the pirate is so far spread out that he leaves almost nothing for the honest plodder. From year to year he becomes more and more bold, and finally, having duped and bulldozed his professional brethren, he turns his attention to lay people, before whom his hideous hypocrisy is supported by consummate strategy. He wants to raise funds for a ward 'for the poor, you know,' run *soirées*, parties, etc., until a necessary sum is wrung out of the pockets of the unsuspecting. Then he suddenly blooms out a specialist, and now that he is safely entrenched behind his own walls, in his zeal for patients his audacity is villainous, for through the mails, he is a constant intruder, incessantly appealing to practitioners to send to him that which they themselves depend upon for their living.

"Let every independent, self-respecting member of the profession rise and stamp out this scorpion. He is the bane, the curse and blight of a profession which without him has a career of usefulness and prosperity before it."

